# Chronic Stress Contributes to Osteosarcopenic Adiposity via Inflammation and Immune Modulation: The Case for More Precise Nutritional Investigation

**DOI:** 10.3390/nu12040989

**Published:** 2020-04-02

**Authors:** Jasminka Z. Ilich, Jennifer C. Gilman, Selma Cvijetic, Dario Boschiero

**Affiliations:** 1 Institute for Successful Longevity, Florida State University, Tallahassee, FL 32306, USA; 2 Independent Researcher, Pataskala, OH 43062, USA; jgilmanster@gmail.com; 3 Institute for Medical Research and Occupational Health, 11000 Zagreb, Croatia; cvijetic@imi.hr; 4 Biotekna^®^, 30020 Marcon-Venice, Italy; dario.boschiero@biotekna.com

**Keywords:** chronic stress, low grade chronic inflammation, osteosarcopenic adiposity, osteosarcopenic obesity, nutrition, micronutrients

## Abstract

Chronic stress and low-grade chronic inflammation (LGCI) are key underlying factors for many diseases, including bone and body composition impairments. Objectives of this narrative review were to examine the mechanisms by which chronic stress and LGCI may influence osteosarcopenic adiposity (OSA) syndrome, originally named as ostoesarcopenic obesity (OSO). We also examined the crucial nutrients presumed to be affected by or cause of stress and inflammation and compared/contrasted them to those of our prehistoric ancestors. The evidence shows that stress (particularly chronic) and its related inflammatory processes, contribute to osteoporosis, sarcopenia, and adiposity ultimately leading to OSA as a final and most deranged state of body composition, commencing at the mesenchymal cell lineage disturbance. The foods/nutrients consumed by modern humans, as well as their altered lifestyle, also contribute to stress, LGCI and subsequently to OSA. The processes can also go in opposite direction when stress and inflammation impact nutritional status, particularly some micronutrients’ levels. While nutritional management of body composition and LGCI have been studied, the nutrients (and their quantities) most affected by stressors and those which may act toward the alleviation of stressful state, ultimately leading to better body composition outcomes, need to be elucidated.

## 1. Introduction

It is well recognized that causes of stress may be of different types, e.g., psychosocial or physical, but each type may precipitate reaction in the brain that activates physiological and hormonal responses leading to adverse health consequences [1,2,3,4], including, but not limited to metabolic, cardiovascular and neuronal. It is evident that no tissue or organ is safe from the negative effects of stress [5]. However, a notable difference exists between acute and chronic stress. Acute stress may induce dynamic adaptation to different demands and is best known for its “fight or flight” (or sometimes “freeze”) response [6]. Chronic stress may have long-lasting maladaptive effects with pathologic consequences on almost every organ-system in the body (immune, nervous, endocrine, cardiovascular) [3], as well as on body composition [4], the relationship with the latter being less studied and understood in humans.

**Figure 1:**
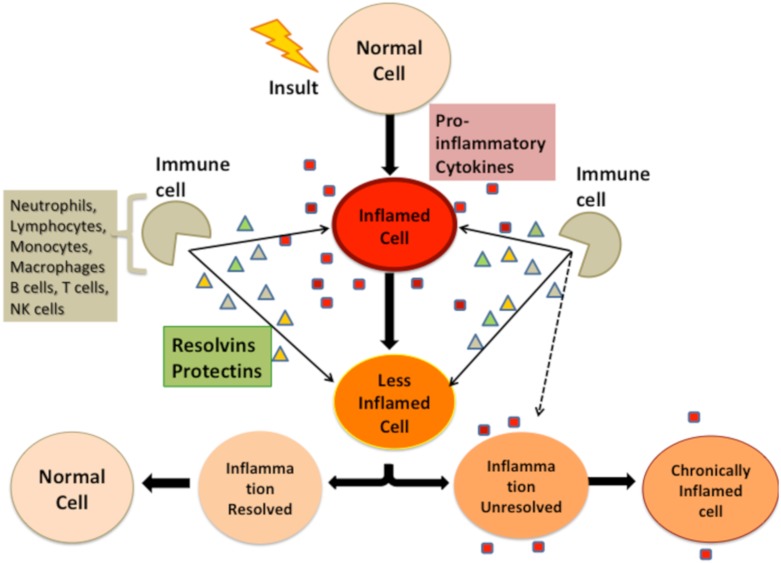
Immune system response to insult leading to low-grade chronic inflammation.

Low-grade chronic inflammation (LGCI) is increasingly recognized as a major underlying cause or promoter of many chronic diseases, including those related to body composition like obesity, osteoporosis [7] and sarcopenia [8] and could be propagated by Western nutritional and lifestyle habits detached from our evolutionary design [7]. It was even coined as “metaflammation” [9] due to its multiple effects on metabolic systems. Although it is difficult to diagnose LGCI clinically as inflammation is at subclinical levels [10], it ultimately is defined as elevation in circulating pro- and anti-inflammatory cytokines (by 2–4-fold), or the continuous presence of specific immune cells in circulation [7,11], as depicted in Figure 1, suggesting a deregulation of resolvins and protectins. In general, chronic stress and LGCI seem to go hand-in-hand, perpetuate each other, and either start or worsen almost every harmful metabolic process leading to ill health and chronic diseases; particularly accentuated in the ever-growing elderly population.

Excess adiposity (body fat/obesity) is inflammatory; inflammation results in accumulation of more body fat due to a positive feedback loop [7,12,13], indicating that body fat plays a major role in LGCI. Compared to our ancestors, body fat has increased and muscle mass has decreased to the point where only elite athletes resemble the body composition of our stone age ancestors—ultimately considered as ideal [14]. Eaton and Eaton estimate that in modern times, on average fat mass has increased from 10% in males and 20–25% in females to >25% in males and >35% in females, while muscle mass has decreased from 50% to <40% in males and from 35–40% to <30% in females [15]. This suggests that modern humans are already at risk of sarcopenia as peak muscle mass is not achieved. Although the lifestyle between our Paleolithic ancestors and modern humans is characterized by vast differences, it provides an evolutionary benchmark reflecting a way “how nature/evolution designed us”. Dietary differences are one factor, discussed later, however, metabolic flexibility and diminished ability to shift metabolic pathways quickly in modern humans is a more recent approach in metabolic/nutritional omics [16]. For example, Freese and colleagues hypothesize that as a result of the remarkably different diet and lifestyle, modern humans are less metabolically flexible compared to our ancestors, especially related to the suppression of ketone production and β-oxidation [17]. Although it is well known that obesity leads to insulin resistance (IR), via LGCI [18], new findings in mice suggest that IR precedes inflammation in adipose tissue [19], suggesting a positive feedback loop between IR and inflammation. While acute IR may be part of the normal response to injury or infection, as seen in the critically ill with associated poor outcomes [20], chronic IR, as seen in type 2 diabetes, may have more serious and long-term health consequences [21]. It is speculated that the current shift in body composition (higher fat mass, lower muscle mass), compared to our Paleolithic ancestors, may be predisposing modern humans to IR, implying that the ratio of adipokine signaling to myokine and osteokine signaling is much higher in modern humans [14].

With this understanding of the relationship of chronic stress, LGCI and adiposity, it seems that the processes feed into and propagate each other, magnifying the risk of developing adverse health outcomes. We present here the hypothesis that chronic stress and its related inflammatory processes, contribute to osteoporosis, sarcopenia and adiposity (either to each individual condition or to a triad; aka osteosarcopenic adiposity (OSA)—previously named osteosarcopenic obesity (OSO) in modern humans by: (a) Increasing/sustaining low-grade chronic inflammation and IR; and (b) shifting mesenchymal stem cell (MSC) lineage commitment toward decreased osteoblastogenesis and myogenesis and increased adipogenesis. We also compare and contrast some of the foods/nutrients consumed by modern humans contributing to stress, inflammatory processes and subsequently to OSA, with the diet of our prehistoric ancestors. Finally, we examine the crucial nutrients known so far to be affected by or cause of stress and inflammation.

## 2. Methods

This is a narrative review utilizing established knowledge and new findings about the topics. Since several important issues are discussed in this manuscript, each of them complex enough to stand by on its own, we believe that the narrative review gives a better overview of the topics and their interactions. At the same time, this being a narrative review presents a certain limitation to the manuscript. The literature searches were conducted in PubMed and Google Scholar, as well as within government and professional/health organizations’ publications in English language, without particular year/date boundaries and to include adults of both sexes. All authors first screened the referenced abstracts for relevance with the ensuing follow up of the complete article for the inclusion of pertinent information. We focused on the following broader areas: Chronic stress, low-grade chronic inflammation, and body composition. Literature searches were performed using combination of key-words and the use of Boolean AND, to include chronic stress with inflammation, immune system, chronic diseases, adiposity/obesity and nutrition. The most recent review and update on the osteosarcopenic obesity diagnostic criteria, characteristics and treatment principles was published in 2019 by Kelly et al. [22]. Therefore, the references from that paper were explored and the phrase “osteosarcopenic obesity” was used in the search to capture any relevant articles published in the meantime.

## 3. Hypothalamic–Pituitary–Adrenal (HPA) Axis

**Figure 2:**
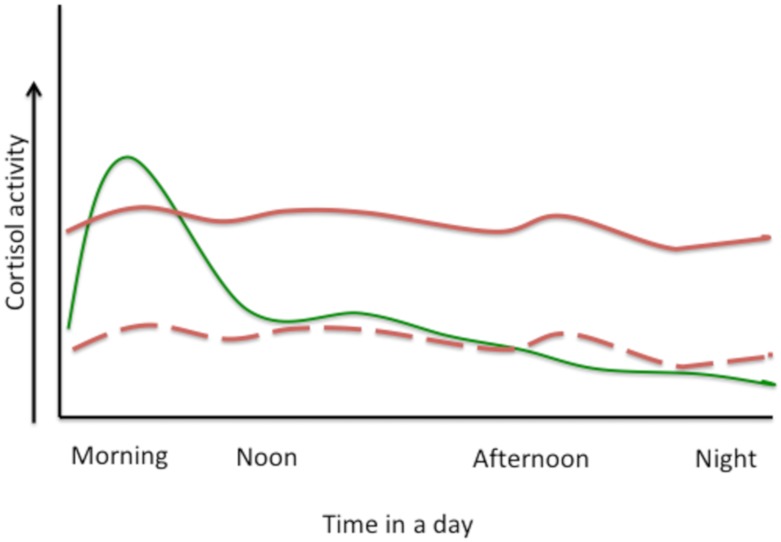
Hypothetical presentation of cortisol activity in normal diurnal variation, full green line (with slight ups and downs during eating or other activities) and under chronic stress, full red line (flat-high, when it is lower than normal-morning and higher than normal-night) or dashed red line (flat-low, when it is lower all the time).

The HPA-axis has a central role in regulating many homeostatic systems in the body; metabolic, cardiovascular, immune, reproductive, to name just a few, and it connects the central nervous system with peripheral sites of immune response and inflammation [1]. HPA-axis activation has a crucial role in the response to stress, however different types of stressors (e.g., psychosocial vs. physical) or kind of stress (e.g., acute vs. chronic) activate HPA-axis via different pathways [1]. Acute stress will cause a surge in catecholamines secretion from the adrenal medulla and release of cortisol from the adrenal cortex, resulting in a quick burst of energy, heightened cognitive functions, increased immunity, lower sensitivity to pain, and higher blood pressure, all aimed for survival [23]. After stressors subside, stress-hormone release declines, mostly via negative feedback on HPA-axis by cortisol, and homeostatic equilibrium is achieved. Chronic stress however, results in flat-high diurnal cortisol release (with lower-than-normal levels in the morning and higher-than-normal levels in the evening) or flat-low levels, both resulting in abnormal level of daily serum cortisol concentration [24]. Figure 2 depicts hypothetical normal diurnal levels of cortisol and those under chronic stress.

The immune system is highly sensitive to glucocorticoids (cortisol) and the most profound consequences of increased glucocorticoid secretion caused by HPA-axis activation are development of the resistance of glucocorticoid receptors located on immune cells. This may lead to immuno-pathology with increases in local and/or systemic inflammatory mediators, like nuclear factor kappa-beta (NF-kB), tumor necrosis factor (TNF)-alpha, and interleukins (IL-1, IL-6) [24]. In other words, immune cells become insensitive to regulatory control of cortisol resulting in the increase of inflammation. Eventually this situation leads to many physiological and mental disorders, including cardiovascular problems, diabetes and depression [25,26,27]. Glucocorticoid resistance, possibly separate from primary-generalized-glucocorticoid resistance [28], is now recognized as a key complication of inflammatory diseases [29]; however, little is known regarding its role in the impairments of body composition, namely in worsening bone and muscle health and increasing adiposity, particularly visceral fat accumulation and infiltration of fat into bone and muscle.

## 4. Bidirectional Relationship between Stressors and Products of Immune Response

Typically, when the HPA-axis is activated by stressors (of which could also be the inflammatory products) high levels of glucocorticoids are released to suppress the immune response by inhibiting the expression of pro-inflammatory cytokines (e.g., IL-1, IL-6, TNF-alpha) and stimulating the expression of anti-inflammatory cytokines (e.g., IL-10, IL-13) in immune cells. Indeed, the therapeutic use of high doses of corticosteroids, to treat various inflammatory diseases because of their ability to suppress immune system, is common [30]. Therefore, the paradigm existing from 1980s to 2000 was that stress, by activation of HPA-axis and release of glucocorticoids, suppresses the immune system and inflammation. Current understanding however, changed that paradigm toward the interpretation that stress leads not just to suppression but also to hyperactivation of the immune response because immune system is under tight neuroendocrine control, while products of the immune system can affect both central and peripheral nervous systems activity [31]. For example, during an immune response, released pro-inflammatory cytokines (e.g., IL-1) can pass through the blood–brain barrier and interact with neurotransmitters, altering their metabolic activity and leading to the activation of HPA-axis and release of corticotrophin releasing hormone, glucocorticoids, catecholamines, and other products of the stress [31]. As stated nicely by Straub: “Stress induces inflammation that induces more stress that induces more inflammation and so on…. It could also go in opposite direction where the origin is in any pre-existing (or even genetically predetermined) condition, which induces inflammation which then induces stress which then induces more inflammation which induces more stress and so on…” [1].

### Interaction of Stressors and Immune System and Potential Health Outcomes

**Figure 3:**
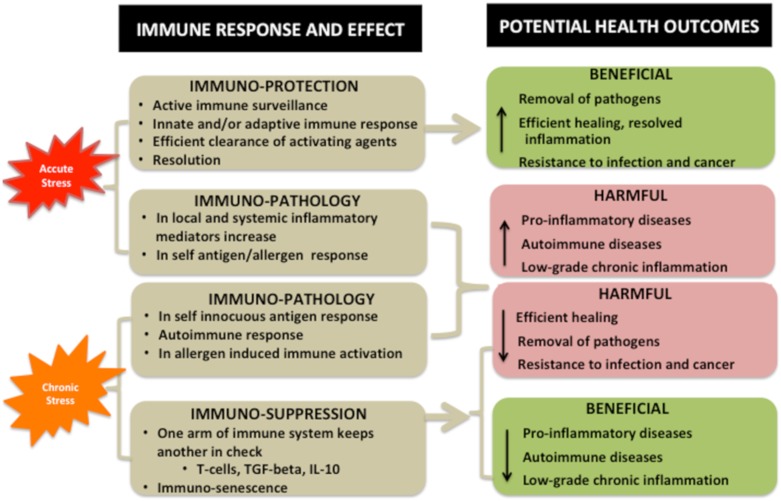
Beneficial vs. harmful effects of stress on immune system and potential health outcomes.

Figure 3 presents beneficial vs. harmful effects of stress on immune system and potential health outcomes. It is important to note that acute stress may have both immuno-protective and immuno-harmful effects resulting in either beneficial or detrimental consequences on health [32]. For example, short-term, acute stress will rapidly activate immune surveillance and innate and/or adaptive immune response, followed by efficient clearance of activating agents and resolution of both stress and inflammation. The health benefits will be evident in the removal of pathogens, efficient healing, resolved inflammation, and possible prolonged resistance to infection and cancer (by the resulting antigen formation). Another example of the positive role of inflammation is in skeletal muscle regeneration [33]. Immuno-harmful effects of acute stress may be reflected in self-innocuous antigen formation, autoimmune response and in allergen induced immune activation. This might cause increased risks for pro-inflammatory and autoimmune diseases and rise in and/or maintenance of LGCI. For the comparison, it is reasonable to assume that the stress experienced by our distant ancestors was primarily of acute nature [17].

Consequences of chronic stress are more complex, yet subtle and sometimes require years or decades to show some pathological effects. Chronic stress can have a multitude of effects which are not limited to one organ. The brain is also susceptible to the effects of chronic stress, resulting in inflammation and increased immune cell recruitment [34]. Immuno-harmful effects may up-regulate local and systemic inflammatory mediator production, resulting in non-resolving inflammation, or even autoimmune disease (e.g., arthritis, lupus) or worsen asthma and allergies [34], as well as contribute to obesity, cardiovascular disease and depression. Immuno-suppressive responses may involve immune cells that inhibit other immune cells, suggesting that suppression of immune system can occur via specific immune cells or messenger molecules that reduce the function of other immune cells [35,36,37]. It is well known that regulatory T cells, IL-10, and TGF-beta can be immuno-suppressive, ultimately acting in a self-regulatory capacity and resulting in immuno-senescence [37]. However, an issue may be that these immuno-suppressive factors inhibit anti-tumor activities resulting in a negative cancer prognosis [38]. Broadly speaking, suppressing the immune system can decrease resistance to infection and pathogen removal, and be oncogenic. Conversely, improving the immune system may decrease healing time and reduce pro-inflammatory/autoimmune diseases, and LGCI (Figure 3).

## 5. Osteosarcopenic Adiposity (OSA) as a Model for Studying and Monitoring Changes in Body Composition

When it was first identified in 2014 [12] and coined as osteosarcopenic obesity (OSO), it was described as a syndrome in Caucasian aging women [39,40] and linked to their lower functional performance [41]. Subsequently, it has been recognized and studied throughout the world in different populations, including Koreans [42,43,44,45], Chinese [46,47,48], Mexicans [49], Brazilians [50,51], Italians [52], and most strikingly, it was even identified in Greek young (18–20 years) obese men and women [53]. Other studies have linked OSO with various other ill consequences, including cancer and kidney disease; see review [22].

As it was addressed in the recent 5-year update on OSO, the current working definition of OSO is that it is a condition encompassing the simultaneous deterioration of bone (osteopenia/osteoporosis) and muscle (sarcopenia/dynapenia) with the increased presence of fat (adipose tissue), as either overt overweight/obesity or redistributed fat (in visceral regions), as well as infiltrated fat into bone and muscle [22]. For a more widespread interpretation, body composition includes bone, muscle (or lean tissue as its proxy) and adipose tissues. There are accepted criteria to diagnose osteopenia/osteoporosis and sarcopenia (the latter with a few favored working definitions); however, the main shortcoming of OSO as a concept is that there is still no global consensus on diagnosing obesity or the amount of adipose tissue in the body, apart from BMI, which is overwhelmingly restricted for those purposes due to its multiple limitations [22]. The reasons for difficulties in diagnosing obesity (and reaching consensus) are due to the high level of heterogeneity of adipose tissue which encompasses visceral, subcutaneous, ectopic (infiltrated in liver, heart, bone, muscle) fat, as well as white, brown, and beige fat cells.

The heterogeneity of adipose tissue is becoming increasingly recognized and was recently addressed [52,54,55]. In that view, Perna et al. suggest that osteosarcopenic obesity be regarded as two different phenotypes: Osteosarcopenic visceral obesity and osteosarcopenic subcutaneous obesity [53]. In their study in 801 bedridden hospital patients >65 years, the osteosarcopenic visceral obesity was more prevalent than osteosarcopenic subcutaneous obesity and was associated with worse metabolic health, inflammation and a higher risk of fractures [52]. This suggests that visceral obesity may be more common and more dangerous within the OSO than the subcutaneous obesity. Within the sarcopenic obesity, Alalwan [54] argues that the main reason for the loss of muscle strength and mass is the increase in ectopic fat in muscle, or peri-muscular adipose tissue (not subcutaneous fat) and its increase with age and infiltration into the muscle [54]. Similar outcomes have been argued for the bone and subsequent osteoporotic obesity [12]. Conversely, individuals with sarcopenic obesity may have benefits compared to those with sarcopenia alone [55] or osteoporosis alone [56], due to the “obesity paradox” and possibly higher amount of subcutaneous compared to visceral fat in these cases. For example, a study in over 600 elderly men and women showed that those with sarcopenia only had higher risk of hip fractures and some other health impairments compared to their sarcopenic obese counterparts, although in this study the distinction between ectopic/visceral and subcutaneous obesity was not identified [55].

In view of the recognized features of the heterogeneity of adipose tissue, we propose here a more appropriate name for the osteosarcopenic obesity syndrome—the “osteosarcopenic adiposity” syndrome. It is still the same condition encompassing bone and muscle loss and increase/redistribution of adipose tissue. We propose the change of the name based on the fact that body adiposity term generally includes the body fat of all kinds (which exactly falls into our original definition of osteosarcopenic obesity-adiposity [12]), while obesity refers more to the overweight as typically defined by high BMI.

Although some updated criteria for OSA based on published and validated individual cut-offs for osteopenia/osteoporosis, sarcopenia and obesity have been presented recently [22], it is hard to collate the OSA prevalence in different ethnicities (e.g., Caucasians, Koreans, Chinese, Latin Americans) due to the varied criteria used for its diagnosis. Furthermore, diagnosing OSA may ultimately be specific to different ethnicities, which would be the most reasonable approach given the discussion around adjusting BMI cut-offs for different ethnicities [57]. In view of this, a more important aspect to consider is that OSA, a grave impairment in body composition, is being researched, diagnosed, and possibly prevented/treated in different populations, even within the existing limitations for its global diagnosis. It is obvious that the health consequences of a disease with comorbidities are greater than the sum of its individual components; e.g., diabetes with hypercholesterolemia, hypertension, and impaired kidney function [58]. However, measuring the effect, even in common diabetes comorbidities (hypercholesterolemia + hypertension + kidney disease) is not typical; to some extent, each condition is treated separately. The same may be true for OSA; each individual condition has its associated comorbidities but health outcomes are compounded when all three are present (osteopenia/osteoporosis + sarcopenia + obesity). In other words, the OSA model is ideal for studying, monitoring and possibly treating changes after any internal or external influences affecting body composition, especially considering that those tissues originate from the same stem cell lineage, as discussed below.

### Key Cellular and Endocrine Interactions among Bone, Muscle, and Fat Tissues

From a broader perspective, the scientific and medical communities must begin to recognize the interplay between diseases/conditions to aid in their prevention and treatment. Accordingly, among three tissues of interest here and their confirmed functioning as endocrine organs, we discuss some molecules secreted by bone, muscle and adipose tissues that make the cross-talk network among them. The fate of each tissue may be regulated by various factors depending on its micro and/or macro environment. At the cellular level, the mesenchymal stem cells (MSC), precursors for bone, muscle and adipose tissue (among various others), may change their lineage commitment under different conditions. Their normal regulatory processes may be disrupted by inflammation, stress, obesity, aging and other ill conditions, as well as nutritional factors, ultimately resulting in commitment dysfunction and subsequent, respective tissue impairments. We will briefly discuss here the bone marrow stromal cells, satellite cells and adipose-derived stem cells giving rise to osteoblasts (bone forming cells), myocytes and adipocytes, respectively. A more detailed description has been previously published [12].

The major factor for osteoblastogenesis, as well as for osteoblast maturation and differentiation is runt related transcription factor 2 (RUNX2), also known as the core binding factor alpha-1, along with the musculoaponeurotic fibrosarcoma oncogene homolog (Maf). They both have a slight inhibitory action on myogenesis and hinder adipogenesis. Specifically, the RUNX2 action is enhanced by estrogen and regulated by prostaglanding eicosanoids 2 (PGE_2_) [59,60]. RUNX2 is also essential in shifting the MSC differentiation from adipogenic (slightly favored even under normal condition) to osteoblastogenic milieu. The key signaling molecules regulating MSC toward myogenesis are myogenin and myoD. Most importantly they are promoters of MSC/satellite cells differentiation into myocytes. They are also strong inhibitors of adipogenesis and to a lesser extent osteoblastogenesis [61,62]. Regarding adipogenesis, the nuclear peroxisome proliferator-activated receptor gamma (PPAR-gamma) is an ultimate promoter of adipogenesis and hinders both osteoblastogenesis and myogenesis. The PPAR-gamma is also involved in adipocyte maturation and metabolism [63,64,65].

Among the master cell regulators, the transforming growth factor beta (TGF-beta) is the one that maintains the MSC population, and among other functions, highly promotes osteoblastogenesis and myogenesis, but inhibits adipogenesis [66]. Insulin growth factor-1 (IGF-1), the true anabolic factor, also promotes osteoblastogenesis and myogenesis but reduces adipogenesis [67]. On the contrary, a strong proinflammator, tumor necrosis factor alpha (TNF-alpha), produced by visceral adipocytes, is a major promoter of adipogenesis and reducer of osteoblastogenesis and myogenesis [68].

On the endocrine level, each of the three tissues—bone, muscle and fat, has a special regulatory action on another one with possible systemic effects. For example, osteocalcin, a major protein secreted by osteoblasts, but better known as a bone formation marker, has a role in energy metabolism by regulating insulin sensitivity and glucose utilization, as well as adiponectin secretion by adipocytes [69]. Thus osteocalcin, with its hormonal function, has been termed as the first osteokine [12]. In premenopausal women, osteocalcin was positively related to lean tissue [70] and in men it induced testosterone secretion, suggesting its possible anabolic actions on muscle [71].

Investigating muscle as an endocrine organ has achieved certain gains in recent years and a new term, the adipomyokines, was introduced to describe cytokines common to both muscle and adipose tissue [72]. For example, IL-6, known as a strong pro-inflammatory cytokine in systemic circulation, was also identified as a myokine [13], and when released from the working muscle, it can exert some anti-inflammatory properties and improve glucose utilization, as well as satellite cell proliferation [73,74]. Other interleukins, e.g., IL-7 and IL-8 mediate fatty acid beta oxidation, while IL-15, released by exercised muscle improves glucose utilization, indicting the common feature in providing the link between muscle and fat tissues [75]. A recently discovered irisin-hormonal-system, provides the best example of muscle-fat axis interaction. Irisin, secreted from the exercising muscle, causes the browning of the white fat cell depots converting them into the beige fat cells [76], the latter characterized by increased mitochondrial numbers and the expression of uncoupling protein-1, leading to higher heat formation and energy expenditure, similar to that of brown adipocytes.

The endocrine role of adipose tissue is probably the most studied. Endogenous estrogens formed in adipose tissue by extraglandular aromatization of androgens, constitute the only estrogens in postmenopausal women. Estrogen’s positive effects on bone, muscle and fat are well recognized [77] and its depletion leads to loss of bone and muscle and gain in body fat. The complex actions of adipose-derived adipokines, namely leptin and adiponectin, are still under extensive investigation. Briefly, leptin and adiponectin have the opposing relationship in circulation, have receptors on both osteoblasts and myocytes and have dual effects on bone and muscle, depending on the local or systemic action [78]. For more in-depth review on the issue, please see [12].

## 6. Evolutionary Changes in Lifestyle and Nutrition Contributing to Stress, Inflammation, and Unfavorable Changes in Body Composition

**Figure 4:**
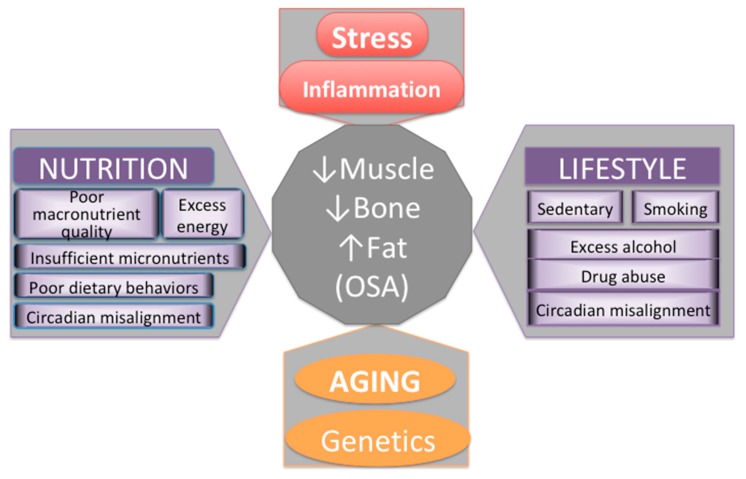
Major contributors to bone and body composition impairments.

Major contributors to body composition outcomes and/or impairments are presented in Figure 4. While there is nothing we can do about our genetic makeup and the inevitable process of aging, there are some nutritional and lifestyle factors that we can modify. The influence of those might not be as powerful, but they can still make substantial difference in how we age, how our body composition develops/changes and how our overall health status emerges.

### 6.1. Misalignments of Circadian Rhythm

All biological systems have evolved synchronized with the natural circadian rhythm of the Earth’s day/night cycle in the way that all physiological and behavioral processes are aligned. The relationship between circadian rhythm and epigenetic changes in immune system, metabolism of nutrients, and different diseases (e.g., cardiovascular, obesogenic, metabolic) has been extensively studied. See review [79]. Moreover, the connection between circadian rhythm and mammalian target of rapamycin (mTOR) signaling pathways (crucial for numerous biological processes and metabolism of bone, muscle, and adipose tissue) has been reported recently [80]. Accordingly, the robust circadian rhythm is necessary for the proper functioning of all body processes. Unfortunately, modern humans’ lifestyle and the way humans organize their sleeping and eating activities (either by choice or necessity) is such that it often leads to violation and major misalignments of circadian rhythm, leading to increased risks for serious metabolic disturbances and diseases of modern time, including obesity, diabetes, hyperlipidemia, and metabolic syndrome [79]. For example, even adolescent girls with evening chronotypes and social jet lag showed greater obesogenic phenotypes, compared to their normal-morning chronotype counterparts, despite that the duration of sleep was taken into account [81].

### 6.2. Diet and Lifestyle of Modern Humans, in Comparison to Our Prehistoric Ancestors, Adversely Affect Metabolic Health and Body Composition

**Figure 5:**
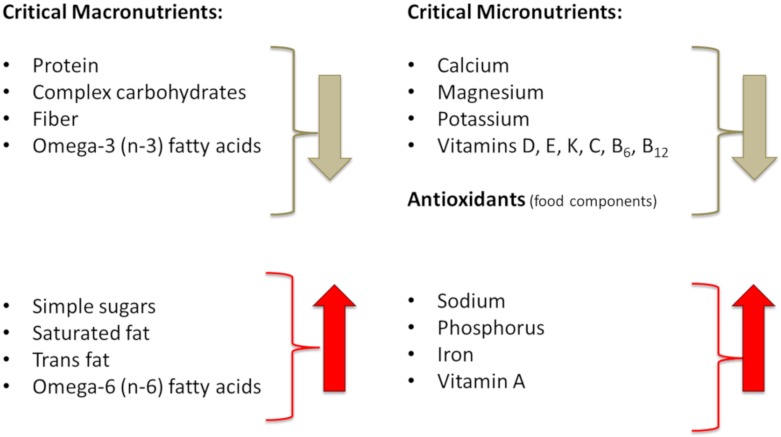
Major nutritional shortfalls or excesses of modern diets compared to our prehistoric ancestors.

The major alterations in dietary intake reflecting the evolutionary discordance between current diet and our ancestors’ diet in Paleolithic period (some 40,000 years ago, when our genetic profile was formed) are considered to be: (a) Overall increased energy intake and decreased energy expenditure; (b) intake of meat and fat high in saturated and trans fatty acids and omega-6 (n-6) polyunsaturated fatty acids (PUFA) but low in omega-3 (n-3) PUFA; (c) low intake of complex carbohydrates and fiber, but high intake of simple sugars and processed cereal grains; (d) lower intake of protein and its lower quality; (e) lower intake of fruits and vegetables; and (f) overall intake of foods with low micronutrient density, leading to lower consumption of potassium, calcium, magnesium, vitamins C, D, E, K, and antioxidants, but higher consumption of sodium and phosphorus [7]. It is worth noting that foods which prehistoric people did not eat represent ~72% of energy of a modern human dietary intake [82]. The foods not available to prehistoric but so common to modern humans include: Dairy foods; cereal grains; refined sugars; refined vegetable oils (including margarines, shortening); salt (except when naturally present in food); and alcohol (except by accidental fruit fermentation) [7,82]. Figure 5 presents the major components of Western-type diet that changed dramatically over the millennia, and are considerably different compared to our ancestors’ diet, possibly contributing to both stress and LGCI. We will focus our discussion on the changes in energy intake, as well as the meat and carbohydrate composition addressing how they contribute to stress, LGCI and then subsequently to unfavorable changes in body composition.

#### 6.2.1. Energy (Calorie) Intake

Obviously, excessive consumption of food, particularly nutrient-low, energy-rich food, provides surplus of energy and body fat accumulation and is one of the causes for global rise in overweight and obesity [83]. However, overeating also triggers the inflammatory pathways [84,85], causing both physiological (from excess food) [86] and psychological (from guilt of overeating or eating bad) stress [87]. As reviewed earlier, overnutrition in modern societies contributes not just to obesity, but also to immunological disturbances, and thus amplifies bad outcomes of the diseases linked with obesity, like osteoporosis, sarcopenia, atherosclerosis, diabetes, and fatty liver disease [7,85]. One mechanism how excess of food (and underlying excess of glucose causing subsequent hyperglycemia) may trigger the inflammatory response is via oxidative stress and formation of reactive oxidative species (ROS) [88]. Another may be the response of the endoplasmic reticulum in adipocytes to stress of overeating and increased demands of subsequent adiposity [86]. In any case, it is now established that inflammation and obesity propagate each other [7], probably via a combination of mechanisms described above.

On the other hand, energy restriction (without malnutrition) has been shown to exert multiple benefits, including metabolic and anti-inflammatory, and extends life expectancy. In addition to multiple studies attesting to this notion as reviewed in a new meta-analysis [89], the most recent study conducted in both humans and mice, reports that short-term and/or intermittent energy restriction and fasting leads to decreased number and activity of circulating monocytes, but does not compromise immunity during acute inflammation [84].

#### 6.2.2. Transformation of Meat and Fat Composition and Changes in Their Intake

Saturated fatty acids (SFA), known for their adverse effects on cardiovascular system [90], are mostly stored in fat components of animal meat that we eat. The meat from wild animals consumed by prehistoric humans was lean and low in fat [91]. However, feeding grains, particularly corn, to animals (started from 1885 in US) [92] changed the meat composition of livestock, resulting in marble meat (excessive triacylglycerol accumulation in muscle), ultimately enhancing its taste and increasing its popularity among modern consumers. This resulted in much higher consumption of SFA, in addition to the overall overconsumption of meat. Based on the newest analysis from NHANES data (1999–2016), more than 10% of daily energy intake comes from SFA, due to high consumption of red and processed meat [93]. Moreover, lean meat consumed from wild animals was not just low in SFA, but it was high in PUFA and monounsaturated fatty acids (MUFA) with n-6/n-3 PUFA ratio of about 1–2, compared to the present Western diet with ratio of n-6/n-3 in the range of 10–20. Dependence on seeds, nuts, greens, and wild plants of prehistoric humans, also contributed to the optimal n-6/n-3 ratio in their diets [94].

The n-6 to n-3 PUFA ratios changed to the worse in almost all populations, compared to that of prehistoric humans. The closest to optimal values (n-6/n-3 of 1–2) was the ratio of the Mediterranean diet prior to 1960. Current ratio in Western type diets (US and Western Europe) is about 15–20 [13,90,95,96]. It has been shown that high levels of n-6 PUFA increase risk for many immune and inflammatory diseases (thrombosis, arthritis, lupus), as well as obesity, osteoporosis and CVD, while those from n-3 family are beneficial for health [7,13,90]. These effects become understandable when examining the metabolic pathways of n-6 and n-3 PUFA series. Overproduction of arachidonic acid, originating from linoleic acid (n-6) rich in fatty meats, oils, and eggs, leads to increased formation of pro-inflammatory eicosanoids (namely, prostaglandins-2, tromboxanes-2 and leukotriens-4), implicated in many inflammatory and autoimmune disorders. On the contrary, metabolism of n-3 series PUFA, e.g., alpha linolenic acid, rich in fish, nuts, berries and greens, releases anti-inflammatory eicosanoids, including prostaglandins-3, tromboxanes-3 and leukotriens-5 [7]. Although the optimal ratio of n-6/n-3 is not confirmed in human studies, or known exactly, it is thought to be 4 or lower [97].

Additionally, partially hydrogenated vegetable oils formed in the production of margarine and vegetable shortening, known as trans fats have adverse effects on CVD due to their raise of low-density-lipoprotein [98] while the reduction of trans fats in diet was associated with a reduction in mortality from heart disease [99]. Trans fat production and consumption was at its peak in the 1960s, which subsequently dropped, yet they still present a danger for human health.

#### 6.2.3. Changes in Carbohydrate Intake

Another dramatic change in the diet of modern humans, in comparison to that of prehistoric period, is a widespread consumption of cereal grains and simple sugars, the latter either in the form of sucrose, high fructose corn syrup (HFCS) or other simple sugars used in the production of modern food or added in drinks. Highly criticized as having deleterious health consequences are sugar sweetened beverages (SSB) and the recommendations are that even 100% fruit juices should be limited in both children and adults [100]. According to the new NHANES data analysis between 1999 and 2016 on some 44,000 adults, 42% of energy in typical American diet comes from refined grains, starchy vegetables and added sugars [90].

The surveys about global and national simple sugar consumption show that the adult consumption in developed countries is up to ~25% of total energy intake [101] and in the US it peaked at 42% of total energy intake in 1998 [102], after which it started declining, reaching some 15% based on the 2008 survey [103]. Despite the reduced trends due to the wide-spread campaigns and health concerns, the overall simple sugar consumption is still relatively high [101], especially in view of the World Health Organization recommendation of a maximum of 10% of total energy and a conditional recommendation of 5% for health benefits [104].

Although a recent review and meta-analysis found no difference in the pro-inflammatory effects from sucrose, fructose (free or as part of HFCS), glucose, or other possible simple sugars added in food [105], the case with fructose may be more complex. Fructose, particularly, promotes de novo synthesis of free fatty acids in liver resulting in metabolites that trigger ROS formation and inflammatory responses. Additionally, some evidence suggests that the excess consumption of fructose, primarily from HFCS may be a risk factor for non-alcoholic fatty liver disease (NAFLD), which is related to metabolic syndrome [106], although other researchers suggest that it is too early to draw conclusions about fructose consumption and NAFLD [107]. It was reported that visceral fat accumulation is higher with fructose intake in the NAFLD patients [108,109]. Visceral fat by itself is the high inflammatory tissue releasing the host of pro-inflammatory cytokines and maintaining the LGCI [7,12]. Additionally, fructose may cause the bacterial overgrowth in gut, increasing its permeability and leakage of endotoxins and lipopolysaccharides triggering the release of pro-inflammatory cytokines [110]. On the other hand, fructose has a lower glycemic index than sucrose (or glucose alone), which might lower some of the post-prandial stress responses of the high sugar diet [111]. Furthermore, fructose is present in fresh fruit and therefore would be considered part of a healthy diet; it even seems higher fruit consumption is associated with a reduced risk for type 2 diabetes [112]. It is possible that excess fructose consumption (not from fruit), contributes to negative metabolic effects when it is a component of an overall unhealthy lifestyle, or metabolic dysfunction is already present.

The link between simple sugars and inflammation is difficult to ascertain, given the complexity in singling out one component of the human diet in addition to other environmental variables [113]. However, it is logical to assume that lowering the intake of simple sugars will have some benefits, such as reducing postprandial glucose and avoiding overstimulation of the insulin response [114]. Additionally, it is important to point out that simple sugars trigger the formation of the advanced glycation end products (AGEs) in food containing fat, protein, and sugars during frying or cooking at high temperatures. AGEs can also form endogenously in the body by glucose auto-oxidation, thus their concentration was found to increase with hyperglycemia. Endogenous AGEs also increase with age and are higher in patients with diabetes, atherosclerosis, kidney disease, and Alzheimer’s disease, exhibiting strong pro-oxidative and pro-inflammatory properties and worsening any of the existing disease [115]. Maier et al showed that among the patients with diabetes, those with diabetic foot ulcers had even higher AGEs concentrations along with the higher associated pro-inflammatory markers [116]. Despite their ability to form endogenously, restriction of foods prone to AGEs formation may lower their overall circulating levels in the body [115]. While simple sugars may trigger inflammatory processes, assuming hyperglycemia is itself inflammatory [117], there is still not enough evidence to pinpoint whether it is a particular sugar, or whether it is the overall excessive energy intake that creates adverse health outcomes [105,113].

## 7. Effects of Chronic Stress and LGCI Magnified by Proinflammatory and/or Inadequate Diet on Osteosarcopenic Adiposity

**Figure 6:**
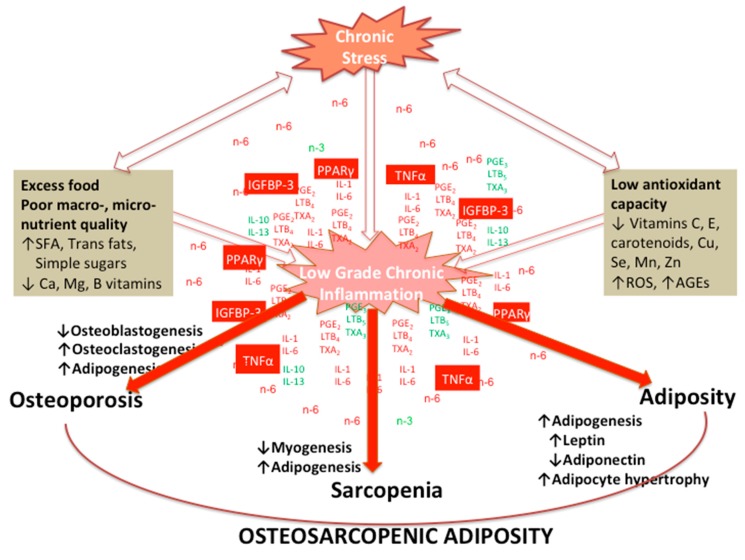
Various factors affecting osteoblastogenesis, myogenesis and adipogenesis, under the influence of stress and low-grade chronic inflammation (LGCI), prompted by poor diets, ultimately leading to disturbance of normal bone, muscle and adipose tissue lineages and development of osteosarcopenic adiposity (OSA).

Figure 6 depicts various factors affecting osteoblastogenesis, myogenesis and adipogenesis, under the influence of stress and LGCI, prompted by poor diets, ultimately leading to disturbance of normal bone, muscle and adipose tissue lineages and development of OSA.

Overconsumption of n-6 PUFA coupled with the underconsumption of n-3 PUFA results in low-grade chronic inflammation and increases susceptibility to osteoporosis, sarcopenia and body adiposity. Other pro-inflammatory dietary factors include excess of food with simple sugars and meat high in SFA and trans fats, as discussed above. Moreover, there are several nutrients of which stress-related deficiency has been linked with some psychological disorders, like depression, post-traumatic stress disorder, and suicidal attempts, as reviewed recently [118]. Besides n-3 PUFAs, these include antioxidant nutrients, like vitamin C, E, carotenoids and selenium, vitamins from the B group (folic acid and B_12_), and magnesium and zinc. The deficiency of those nutrients is implicated in stress-related mitochondrial damage and impaired neurotransmitter signaling. In particular, lower n-3 PUFAs levels in the body affect proper formation of the lipid rafts in the cells of central nervous system leading to neurotransmitter malfunctioning and inadequate formation of serotonin and dopamine [119], causing depression and increased risk of suicide. Antioxidant vitamins along with zinc, selenium, manganese and copper improve antioxidative capacity of the body and reduce reactive oxygen species caused by stress and inflammation [120]. Folic acid and B_12_ contribute to the recycling of homocysteine (toxic to mitochondria) into methionine, while magnesium enhances mitochondrial enzymatic functioning. Deficiency of other B vitamins, namely B_1_ (thiamine), B_3_ (niacin), B_6_ (pyridoxine) has been implicated in impaired neurotransmitter synthesis, along with deficiency of manganese, copper and zinc leading to depression or other psychological impairments [121]. Having adequate status of these nutrients may help with the stress-induced psychological disorders and complement the drug therapies.

Low-grade chronic inflammation from the increased synthesis of pro-inflammatory lipid mediators, prostaglandin E 2 (PGE_2_) and leukotriene B 4 (LTB_4_) by cyclooxygenase 2 (Cox-2) and 5-lipoxygenase (5-Lox), results in an increase in peroxisome proliferator-activated receptor gamma (PPARγ) signaling, producing more adipocytes. The increased adipose metabolism produces more tumor necrosis factor-alpha (TNFα) which increases osteoclastogenesis, decreases osteoblastogenesis and myogenesis and increases Cox-2 expression directly or indirectly via platelet activating factor (PAF). Increased leptin production, also secreted by adipose tissue, can lower bone and muscle formation through its hypothalamic interactions. Furthermore, the elevated PGE_2_ and LTB_4_ result in an increase in insulin like growth factor binding protein-3 (IGFBP-3), which inactivates insulin like growth factor-1 and promotes commitment of MSC to the adipocyte. The net result from these events is an increased susceptibility to OSA, as depicted in Figure 6.

Additionally, in this context of dietary factors affecting body composition via stress and inflammation, human microbiome should not be neglected, being an ultimate consequence of our diet. Although discussion on microbiome is beyond the scope of this paper, particular benefits of amino acid, glutamine—the most abundant amino acid that provides adenosine 5-triphosphate (ATP) demands for cells, on gut microbiota was reviewed recently [122]. Accordingly, glutamine supplementation was found to have protective effect on enterocyte integrity, nitrogen metabolism in enteric bacteria, bacterial translocation and overall intestinal health. Simultaneously, glutamine-mediated cross-talk with gut microbiome improved immune response, and increased resistance to stress, particularly in critically ill patients. Glutamine was also found beneficial in obese and overweight individuals by improving their gut microbiota [123], in skeletal muscle (together with arginine) by reducing inflammation and in bone homeostasis by regulating MSC osteoblastogenesis, as well as exerting immuno-modulatory properties [124].

## 8. Concluding Remarks

Stressful environmental inputs of all kinds may affect neural circuitry via epigenetic changes [125], causing inflammation and biological and organ systems impairments, as well as affecting overall psychological state. We presented the evidence that stress (particularly chronic) and its related inflammatory processes, contribute to osteoporosis, sarcopenia and adiposity ultimately leading to OSA as a final and most deranged state of body composition. The derangement commences at the MSC lineage commitment disruption toward decreased osteoblastogenesis and myogenesis and increased adipogenesis. The foods/nutrients consumed by modern humans, as well as their different lifestyle and misaligned circadian rhythm, all contribute to stress, inflammatory processes and subsequently to OSA. Simultaneously, diet and lifestyle may contribute to either negative or positive mood changes, the former feeding into the stressful state and immune system disturbance, ultimately leading to organ impairments.

The processes can also go in opposite direction when stress and inflammation impact nutritional status, particularly micronutrients’ levels in the body, leading to depletion of calcium, magnesium, zinc, iron and some vitamins, e.g., thiamine, niacin. While recommendations for nutritional management of body composition and LGCI have been studied, the nutrients (and their quantities) which are most affected by stressors and those which may act toward the alleviation of stressful state, are yet to be elucidated.
